# Parotid metastases from primary lung cancer: Case series and systematic review of the features

**DOI:** 10.3389/fonc.2022.963094

**Published:** 2022-08-25

**Authors:** Rulan Wang, Ting Wang, Qinghua Zhou

**Affiliations:** Lung Cancer Center, West China Hospital, Sichuan University, Chengdu, China

**Keywords:** small cell lung cancer, metastasis, parotid, feature, diagnose, treatment

## Abstract

Most parotid metastases have been reported to come from the head and neck; however, cases metastasized from the lung are extremely rare. Missed diagnoses and misdiagnoses occurred quite a few times. Thus, accurately identifying the clinical features of parotid metastasis of lung cancer is important. However, current studies about this issue are mostly case reports, and little is known about the detailed and systematic aspects. We reported three cases of parotid metastases from lung cancer and then systematically searched similar cases through “Pub-Med” and “Web of Science”. Finally, twenty-three patients were included in the study. Eighty-three percent of which were males, and 19 patients were over 50 years old. In all cases with smoking history mentioned, 93% were smokers. The predominant pathological type was small cell lung cancer (SCLC, 13 patients, 56%). Seventeen combined with other site metastasis, while more than half of which were brain metastases. The survival time ranged from 3months-17years, and as for SCLCs, it was only 3months-40months. It can be concluded that clinical features, such as sex, age, smoking history, pathological types, and metastasis patterns, could provide valuable evidence for diagnosis. The lung seems to be the most common primary site of parotid metastases except for head and neck tumors. The two circumstances, SCLC coexisting with Warthin’s tumor and parotid small cell carcinoma with lung metastasis, should be differentiated from parotid metastasis of lung cancer with caution For cases presented as SCLC, more aggressive strategies, such as chemotherapy with immunotherapy and maintenance therapy, may be more suitable. Due to the greater tendency of brain metastasis in such diseases, whole-brain radiation therapy, stereotactic radiosurgery or prophylactic cranial irradiation should be applied to corresponding patients in time. Additionally, lung cancer parotid metastases may be a marker of poor prognosis.

## Introduction

Lung cancer is one of the most common malignant tumors, accounting for the leading cause of cancer death worldwide (1). Pathologically, it can be divided into small cell lung cancer (SCLC) and non-small-cell lung cancer (NSCLC) (2). SCLC, although accounting for only approximately 15%, is extremely malignant (3). Even after effective treatment, the median overall survival is still only approximately 1 year, especially for stage IV diseases (4). Distant metastasis is a major feature of lung cancer, but metastasis to parotid gland is really rare (5). The majority of metastatic malignant parotid diseases originate from head and neck tumors, and as a result, most of the pathological types are squamous cell carcinomas or malignant melanomas. Other pathologic types, such as small cell carcinomas, are rare (6).

Owing to the superficial location, parotid masses frequently appear as the initial symptom of lung cancer parotid metastases. Such situations often lead to parotid gland tumors being misdiagnosed as primary lesions, while ignoring the diagnosis and treatment of the real primary site (7). On the other hand, for the inherent benign impression of parotid tumors, misdiagnosis of metastatic parotid tumors also occurs quite a few, especially when lung cancer presents with parotid mass (8). Therefore, great attention should be given to when parotid pathology reveals an uncommon type. Another concern is that the treatment and prognosis of limited and extensive tumors are different, so accurately identifying features of lung cancer parotid metastases matters. However, current studies about this issue are mostly case reports, and little is known about the detailed and systematic aspects. Here, we reported three cases of parotid metastases from SCLC in our institution and reviewed cases regarding parotid metastasis of lung cancer that published previously, in order to provide some references for the management of such disease. To our knowledge, this is the first study to systematically analyze the characteristics of parotid metastasis in lung cancer.

## Materials and methods

We reported three consecutive cases of parotid metastases from lung cancer treated at our institution and then conducted a literature search with no restrictions on the year of publication. According to the following search strategies: (“bronchial carcinoma” OR “lung carcinoma” OR “lung cancer” OR “lung neoplasms” OR “lung adenocarcinoma” OR “lung squamous” OR “small cell lung cancer” OR “NSCLC” OR “SCLC”) AND (“parotid” OR “salivary”) AND (“metastasis”), the databases “Pub-Med” (http://www.ncbi.nlm.nih.gov/pubmed) and “Web of Science” (https://www.webofscience.com/wos/alldb/basic-search) were fully checked. The latest search date was July 4, 2022. All the identified relevant articles were examined independently by two investigators. Once discrepancies arose, the two reviewers discussed and analyzed the data together and reached a consensus. Studies that did not fit the topic, or were duplicated, or were not full text, or were deficient in clinical information were excluded. In addition, we checked the references within the included studies to avoid any omissions. Since all the articles involved were case reports, the risk bias assessment tool was abandoned in this study. Then, the following information was extracted: the first author, publication year, gender, age, initial symptoms, smoking history, pathological type, primary tumor lesions and size, parotid metastasis lesions and size, other accompanying metastases, treatment, and survival time. Finally, we summarized the above data and analyzed the characteristics.

## Results

### Case Reports

#### Case 1

**Figure 1:**
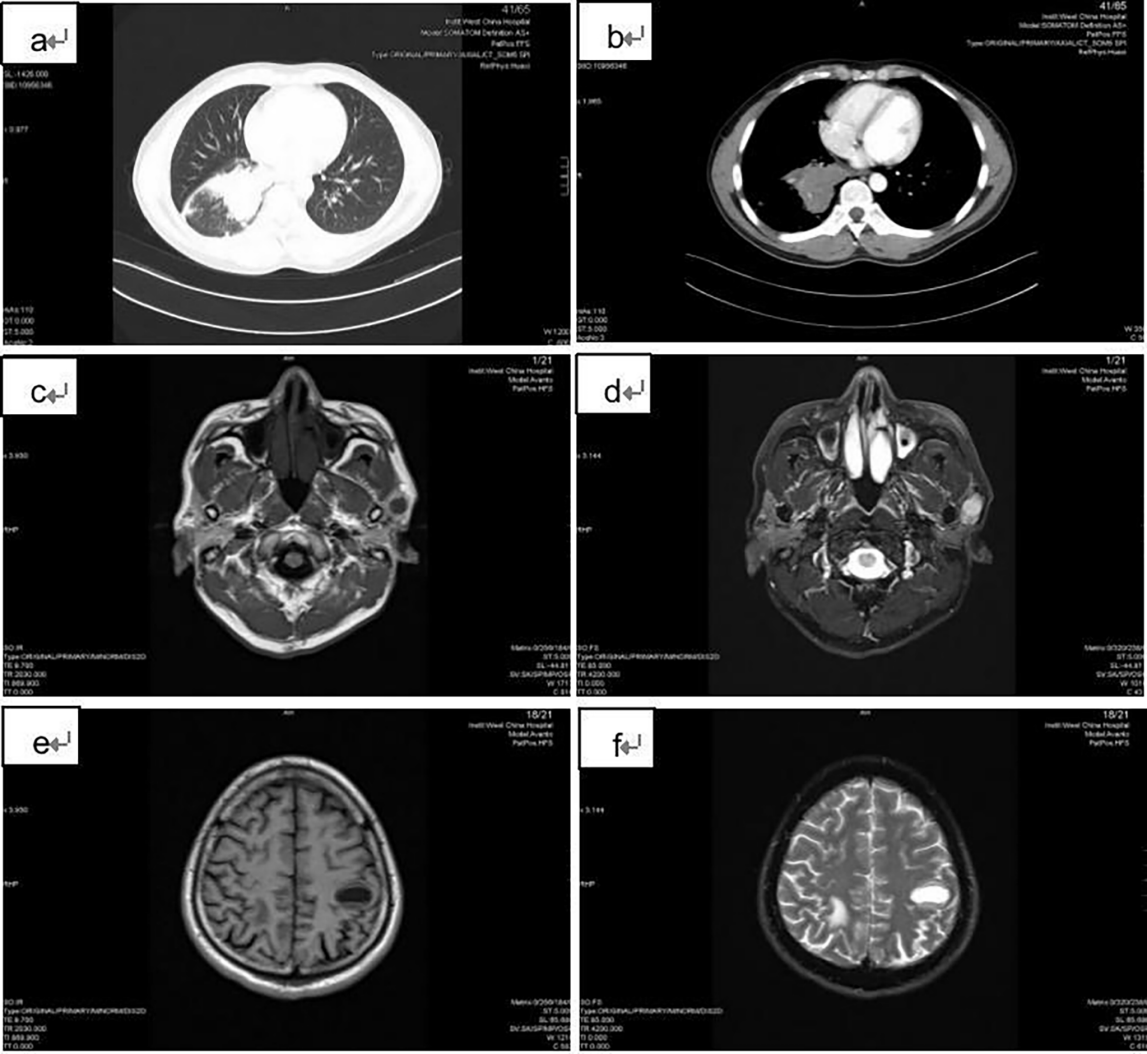
Imaging findings of the patient. **(A)** and **(B)**: the chest CT showed a 6.4 cm x 5.2 cm mass at the right hilar; **(C)** and **(D)**: T1 and T2 weighted head MRI images showed the preauricular mass located at the parotid gland; **(E)** and **(F)**: T1 and T2 weighted head MRI images revealed the brain metastases.

**Figure 2:**
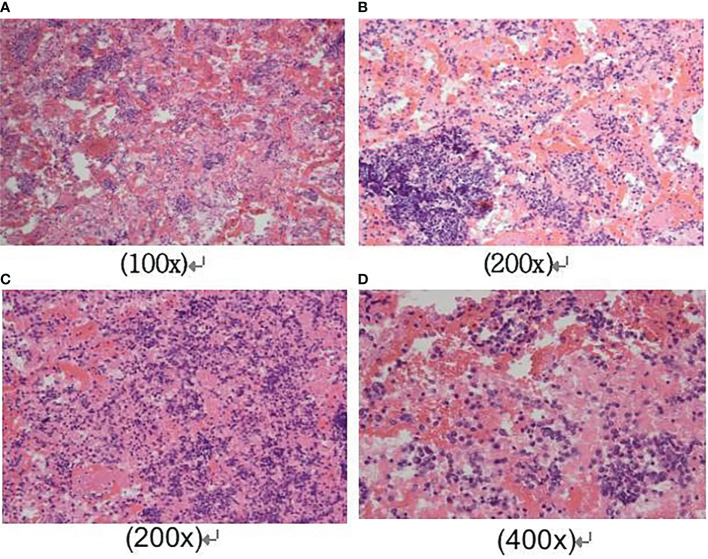
Cytological findings of parotid mass biopsy. Hematoxylin and eosin staining of the fine-needle aspiration biopsy of the preauricular mass revealed a large number of oat-shaped heteromorphic cells with deep nuclear staining at high magnification, distributed in the shape of chrysanthemum nests. The magnification was as follows respectively: **(A)** was 100x magnification; **(B)** and **(C)** were 200x magnification; **(D)** was 400x magnification.

A 42-year-old male came with the painless hard mass that appeared in front of his left ear. He complained that he had little discomfort except occasional cough. Based on the five-year history of heavy smoking, he was arranged for a chest computed tomography (CT), which found a 6.4 cm x 5.2 cm mass at the right hilum (**Figures 1A, B**), suggesting central lung cancer. Subsequent lung biopsy and immunohistochemistry confirmed it was SCLC. Further head magnetic resonance imaging (MRI) showed a preauricular mass located at the parotid gland (**Figures 1C, D**). It is known that tumor metastasis from the lung to the parotid is rare, so there is a big question about the nature of the parotid mass. Hematoxylin and eosin staining of biopsy of the preauricular mass revealed a large number of oat-shaped heteromorphic cells with deep nuclear staining at high magnification, suggesting metastasis of SCLC (**Figures 2A–D**). Additionally, head MRI and abdominal CT showed that the head and the right adrenal were also affected (**Figures 1E, F**). Then, he accepted a standard etoposide and cis-platinum (EP) regimen for 6 cycles, as well as the head and chest radiotherapy. Subsequent reviews showed that the patient entered a partial state of remission. Unfortunately, nine months after the completion of treatment, he died due to the progression of brain metastasis.

#### Case 2

A 61­year­old man presented to our hospital because of persistent cough and confusion about a progressive growth mass at the left parotid. He was a heavy smoker (44-year history of 20 cigarettes per day). The chest and another neck CT showed a large mass at the left hilar and a 2.8 cm x 2.5 cm mass at the left parotid. The lung biopsy confirmed that he suffered from SCLC. Further examination found that the bilateral lung, left axillary, left adrenal and head all had metastatic nodules. The otolaryngology suggested that the parotid mass might be Warthin’s tumor, so it was not examined further. Then, he was included in a multicenter double-blind clinical study (Identifier NCT01450761) and received a standard treatment of etoposide and carboplatin with ipilimumab/placebo. After two cycles of treatment, the tumors were all shrunken, especially the parotid mass, suggesting that the parotid mass was also the metastasis of lung. Due to the previous wrong evaluation of parotid metastasis, the pathological biopsy of parotid gland was not carried out, resulting in the failure to obtain a correct diagnosis. Since the patient has already entered the extensive stage, the choice of treatment scheme has not been significantly affected. However, after four cycles of treatment, the brain metastases progressed, leading to his death. It was only three months since his diagnosis.

#### Case 3

A 50-year-old male underwent the surgery for early left SCLC in April 2012, subsequently underwent 6 cycles of treatment with a standard EP regimen. Then, he followed up regularly. Unfortunately, the recurrence of neck lymph nodes appeared in 2014. Traditional Chinese medicine and chemotherapy (both EP and TP (paclitaxel and cis-platinum) regimen) had little effect. Thankfully, cervical lymph node radiotherapy resulted in a significant mass reduction in March 2015. However, subsequent follow-up found metastatic tumors in the head, parotid and parapharyngeal space. The biopsy of the parotid proved that it was a SCLC metastasis. Then, the patient received a single irinotecan chemotherapy regimen and whole-brain radiation therapy (WBRT). After two cycles of treatment, the tumors remained stable. However, after four cycles of treatment, the patient lost contact with us. It was only less than five months since the parotid mass was founded.

### Literature review

**Figure 3:**
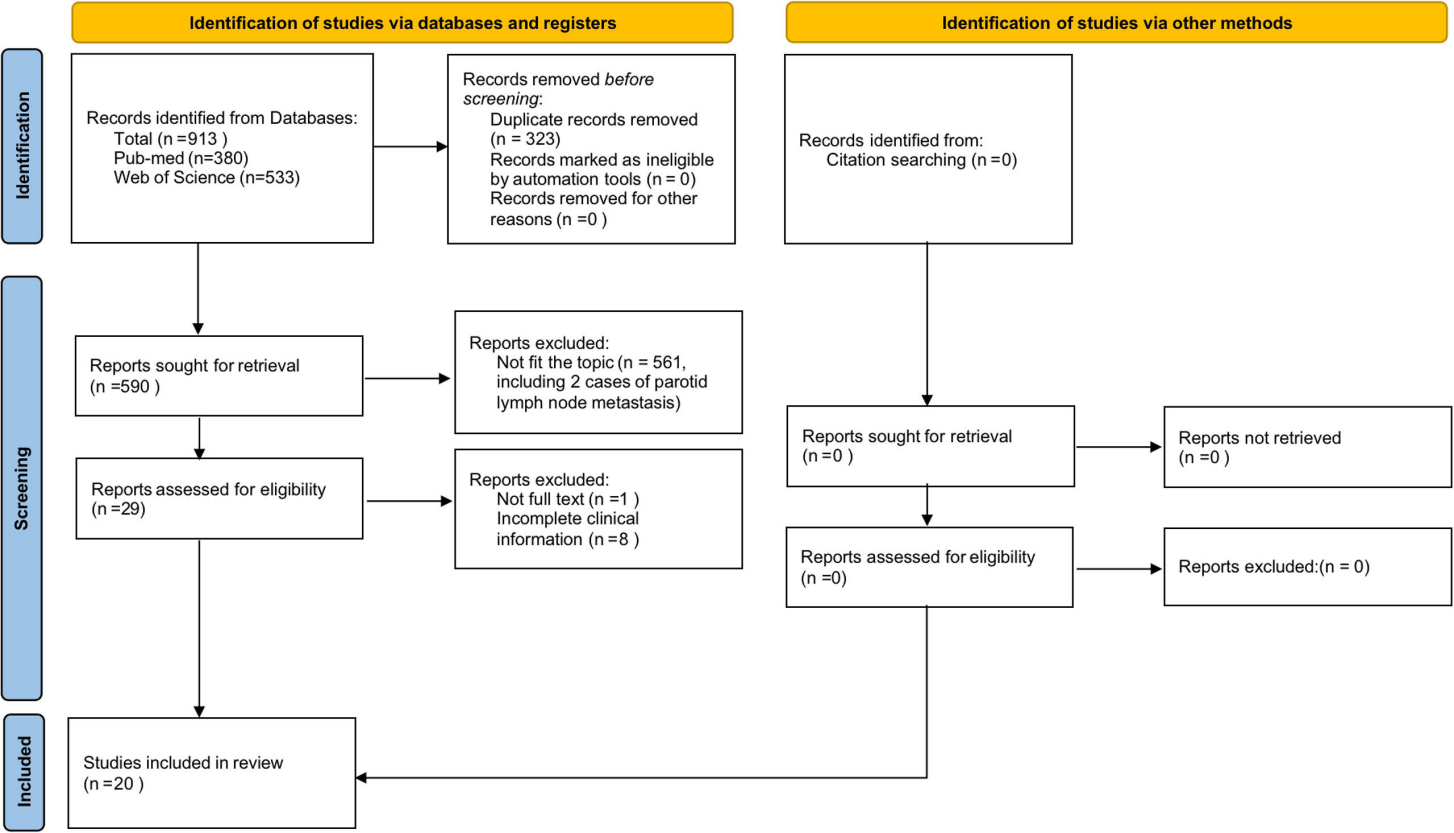
The flow diagram according to PRISMA shows the detailed filtering process.

Then, we reviewed previously published similar cases and summarized the characteristics. We originally identified 913 relevant articles. After removing the 323 duplicate records, 590 were left. According to the exclusion criteria, 562 studies which did not fit the topic [including 2 with parotid lymph node metastasis (9, 10)], 1 without full text, and 7 with deficient clinical information were excluded. Ultimately, 20 articles comprising 20 patients were selected for our study (the detailed filtering process is shown in **Figure 3**). Adding the three patients discovered at our institution, for a total of 23 [**Table 1** (5, 11–26)]. Among the 23 patients, 19 (83%) were males, and 4 (17%) were females. Most patients were over 50 years old (19/23 patients, 83%), with a median age of 59 years old. Fifteen (65%) presented with parotid gland mass as their initial symptom. In all cases with smoking history mentioned, 13 (93%) were smokers and only 1 (7%) was a nonsmoker. The predominant pathological type was SCLC (13 patients, 56%), followed by adenocarcinoma (7 patients, 30%), and squamous carcinomas (3 patients, 13%). Ten of the primary sites were left lungs, and thirteen were right lungs. Twelve (52%) cases presented with left parotid metastasis, 8 (35%) cases with right parotid metastasis, 2 (9%) cases with bilateral parotid metastasis, and 1 case not mentioned. Seventeen (74%) had other site metastases, while more than half (9 patients) had brain metastases. Most patients received chemotherapy, a few combined with radiotherapy or parotidectomy, and one patient obtained lung surgery for misdiagnosis of the parotid tumor. The survival time ranged from 3 months-17 years, and for SCLCs, it was only 3 months-40 months. The above features are summarized in **Table 2**.

**Table 1:** the literature review of all cases of the parotid metastasis of the lung cancer.

Author/year	Age/sex(years)	Initial symptoms	Smoking	Pathological type	primary tumor(cm)	Parotid metastasis tumor (cm)	Other metastases	Treatment	Survival
MF/2004 (5)	72/M	parotid masses	yes	SCC	left apical lung (NM)	left side (3x4)	No	radiotherapy	3 years
Boeger et al/2005 (11)	54/M	right parotid swelling	NM	SCLC	Left lung (NM)	right side (NM)	Left adrenal	parotidectomy	NM
Cantera et al/1989 (12)	40/M	bilateral painless parotid masses	yes	SCLC	main left bronchus (NM)	bilateral side (5x4;2x1.5)	bone	chemotherapy	4months
Yu et al/2018 (13)	64/M	peripheral facial paralysis	NM	SCLC	right upper, middle lobes (NM)	right side (2x2)	liver	Parotidectomy, chemotherapy	>10 months
Katsurago et al/2006 (14)	72/M	NM	NM	AC	right upper and left lower lobes (NM)	Left side (NM)	Adrenal, brain	parotidectomy, radiotherapy, chemotherapy	17 years
Lawande et al/2017 (15)	52/M	facial swelling, chest pain	yes	SCLC	right upper lobe (8.7x6.5)	right side (3.5x3)	Left subdiaphragmatic, adrenal	Radiotherapy, chemotherapy	NM
Lenouvel/2006 (16)	59/M	right preauricular swelling	yes	AC	Right lung (NM)	right side (2x2.5)	renal and multiple bony metastases.	No	deteriorated rapidly
Shalowitz et al/1988 (17)	54/M	facial weakness, dry cough	yes	SCLC	Left hilar and left lower lobe (NM)	Left side (0.5x0.5)	Liver and adrenal	Radiotherapy, chemotherapy	> 3 months
Shi et al/2006 (18)	61/M	right parotid swelling	yes	SCLC	right upper lobe (6.3x5.4)	right side (1.3x1.3)	No	Parotidectomy, chemotherapy	4 months
Ulubas/2010 (19)	59/M	right parotid mass	NM	SCLC	Right lung (9)	right side (NM)	Liver, bone	chemotherapy	10 months
Debnath/2015 (20)	50/M	NM	NM	AC	left lung (NM)	right side (2.5x2)	NM	NM	NM
Hisa et al/2010 (21)	61/M	left parotid swelling	NM	SCLC	right lung (NM)	bilateral side (NM)	No	parotidectomy, radiotherapy, chemotherapy	17 months
Takats et al/2010 (22)	48/F	left parotid swelling	NM	SCLC	right hilar (NM)	left side (3x2)	Pituitary gland, lumber spinal cord	Radiotherapy, chemotherapy	13 months
Norton et al/2020 (23)	65/F	Breathlessness, left parotid swelling	NM	AC	right lung (NM)	left side (NM)	large intra-abdominal lymph node	Refuse treatment	NM
Elena et al/2020 (24)	65/M	left parotid gland mass	yes	SCLC	left lower lobe (5)	left side (2.4)	Brain, cervical vertebrae	chemotherapy	3 months
Yang et al/2017 (25)	66/M	blood-stained sputum, pain near left ear	yes	AC	left upper lobe (4.6)	left side (1x1)	No	Lung cancer operation, chemotherapy	>6 months
Wang et al/2016 (26)	56/F	Parotid swelling, intracranial hypertension	NM	SCC	left upper lobe (3.4x3.3)	right side (2.4x2.4)	Brain	chemotherapy	NM
Sankalp et al/2020 (27)	60/M	Parotid and scalp swelling	yes	SCC	Right upper lobe (5.6x5.4)	right side (3.1x2.6)	Scalp	Chemotherapy, radiotherapy	9 months
Claire et al/2021 (28)	48/F	Parotid swelling	yes	AC	Right lower lobe (2.3x1.5)	left side (2.5)	Brain, retroperitoneal	Parotidectomy, brain metastases resection	NM
NA et al/2022 (29)	79/M	No symptom	yes	AC	Right upper and lower lobe left lower lobe (<3)	left side (1.5)	No	radio-therapy, Parotidectomy	2 year
Present case 1	42/M	left parotid mass	yes	SCLC	right hilar (6.4x5.2)	left side (1.5x1.5)	Brain, adrenal	Chemotherapy, radiotherapy	13 months
Present case 2	61/M	Cough, left parotid mass	yes	SCLC	left hilar (NM)	Left side (2.8x2.5)	lung, axillary, adrenal, brain	Chemotherapy	3 months
Present case 3	50/M	No symptom	No	SCLC	Left lung (NM)	Left side (NM)	Brain, arotid	Chemotherapy, radiotherapy	40 months

M, male; F, female; SCLC, small cell lung cancer; SCC, squamous cell carcinoma; AC, adenocarcinoma; NM, not mentioned.

**Table 2:** Summary of the characteristics of lung cancer parotid metastases.

total	sex	Age (years old)	smoking	Pathological type	Parotid metastasis relative position	Combined with other site metastasis	Survival
23	M:19 F:4	Rang:40-79 Average:58.2 Median: 59 ≥50: 19	Yes:13 No:1 NM: 9	SCLC:13 AC:7 SCC:3	Ipsilateral :12 opposite:8 Bilateral:2 NM:1	Total: 17 brain metastasis: 9	3months-17years (SCLC:3months-40months)

M, male; F, female; SCLC, small cell lung cancer; SCC, squamous cell carcinoma; AC, adenocarcinoma; NM, not mentioned.

## Discussion

Metastatic malignant parotid diseases, accounting for only 6-8% of parotid tumors (30), mostly originate from head and neck tumors, while non-head and neck parotid metastases may originate from the gastrointestinal tract, breast, pancreas and lungs (6). Lung cancer metastasis to the parotid gland is particularly rare (5). Due to the superficial location and benign impression of the parotid (31), missed diagnosis of primary tumors or misdiagnosis of parotid metastasis tumors occurs as common (7, 8). However, there is no appropriate way to avoid the above problems thus far. With regard to different properties and stages, the treatment and prognosis of tumors are also different. To achieve better diagnosis, differential diagnosis, and more effective treatment, we analyzed the characteristics of such patients and attempted to provide reference opinions on their management.

### Diagnosis

As shown in **Tables 1**, **2**, this disease seemed more likely to occur in elderly smoking men in general, which was consistent with the prone crowd of lung cancer (32). Most patients with parotid metastasis of lung cancer had no obvious pulmonary symptoms (5, 12–15, 21–23, 25, 27). The initial clinical manifestation was always a rapidly expanding parotid gland mass, with or without pain, but usually did not invade the skin, sometimes accompanied by facial paralysis, suggesting no specificity. Imaging examination, especially MRI, could provide evidence for the differentiation of benign and malignant tumors, such as boundary and surrounding infiltration (33). Fine needle biopsy is the most commonly used diagnostic technique (7, 34). Usually, the discovery of an unusual pathological type is the initial cause of the suspicion of metastatic parotid tumor. According to the literature reports, parotid metastases can originate from the gastrointestinal tract, breast, pancreas and lungs (6), but the lung seems to be the most common primary site except for head and neck tumors (6, 7) (34, 35). Therefore, when secondary parotid malignancy is suspected and primary lesions are not found at the head and neck, the possibility of lung origin should be considered first. Indeed, positron emission tomography CT may be a pretty choice (29).

### Differential diagnosis

Due to the rarity of parotid metastases of lung cancer, differential diagnosis should be made carefully. The following situations should be handled with caution: (1) Lung malignancy with parotid gland benign tumor, especially Warthin’s tumor, a common benign tumor of the parotid. The majority of parotid gland tumors are benign, but the 2-[18F]-fluoro-2-deoxy-d-glucose (FDG) uptake values of some types, such as warthin’s tumor and pleomorphic adenoma, are pretty high, and even equivalent to that of salivary gland malignant tumors (31, 36). As a result, the misdiagnosis of secondary parotid malignancy occurs frequently. Furthermore, because smoking is an identical risk factor for lung cancer (2) and Warthin’s tumor (37), lung cancer coexisting with Wartin’s tumor is not puzzling (38–41). It is reported that there is a significant correlation between the occurrence of parotid gland warthin’s tumor and lung cancer (38, 42). About 19% of patients with warthin’s tumor in parotid gland also have lung cancer (38). False recognition of the nature of parotid tumors can lead to different tumor stages and treatment. For example, present case 2 mistook parotid metastasis as Warthin’s tumor, and the case of Yang et al. (25) reported accepted the lung cancer operation owing to the wrong judgment of the parotid mass nature. Therefore, accurately discriminating the characteristics of parotid gland masses is particularly important. Parotid benign tumors have a long course of disease and develop slowly (31). Moreover, parotid benign tumors are usually located in the superficial lobe of the parotid gland (43), with no surrounding tissue infiltration and a clear border (31). In contrast, parotid malignant tumors usually grow rapidly (31). Significantly, they are usually discovered at the deep lobe or across the superficial and deep lobe (43), with invasion of the facial nerve or surrounding tissues, and have unclear boundaries (31). Emerging imaging technologies, such as apparent diffusion coefficient (44), diffusion-weighted imaging (45), and dynamic contrast-enhanced magnetic resonance imaging (46), can provide effective help. Therefore, a detailed history and imaging studies are essential for the differential diagnosis. However, the above characteristics still cannot accurately distinguish lung cancer parotid metastasis and lung cancer coexisting with Warthin’s tumor. Thus for patients with lung mass and parotid gland mass at the same time, it is very important to perform pathological biopsy for both. (2) Primary malignant tumors of the parotid gland, especially parotid gland small cell carcinoma (PGSmCC), with lung metastasis. In the current study, small cell carcinoma accounted for the priority (65%) of lung cancer parotid metastases. Distinguishing the parotid metastases of SCLC from lung metastases of PGSmCC is the key diagnostic challenge. Primary PGSmCC, accounting for less than 1% of salivary tumors (47), has a 5-year survival rate of 37%, which is much better than that of SCLC (48). Pathologically, primary PGSmCC can be divided into ductal type, Merkel type and pulmonary type. For the ductal type and pulmonary type, cytokeratin 20 (CK20) staining is negative, but for the Merkel type, CK20 staining is strongly positive (49). In general, Servato et al. suggested that approximately 79% of PGSmCCs were CK20 positive (47). However, SCLC has no ductal morphology, and CK20 expression is negative (50). In view of the above, SCLC with parotid metastases and PGSmCC with lung metastases can be preliminarily differentiated. However, there is still no reliable method to distinguish pulmonary-type PGSmCC from SCLC parotid metastasis because the immunophenotype of the two diseases widely overlaps (50). The order of tumor occurrence may be helpful to solve this dilemma. On the other hand, a study comprising 344 cases of primary PGSmCC observed that distant metastasis in such disease was rare (rate 7.3%) (48). However, for SCLC, distant metastasis is universal. Moreover, there is no literature report of lung metastasis from PGSmCC at present. Regardless, either of the two situations should be treated more radically.

### Treatment

According to the study, many patients (75%) had metastases in multiple parts of the body. Shi et al. (18) reported that this phenomenon implies the disease has progressed to extensive stage, which is a reason for the poor prognosis (32). In particular, SCLC is more malignant than any other pathological type of lung cancer (4). Thus, the survival is rarely more than one year. Therefore, for such patients, more aggressive treatment strategies should be adopted. Chemotherapy combined with immunotherapy has brought the treatment of extensive SCLC into a new era (51, 52). Many studies have confirmed that such a treatment strategy can prolong survival by 2-3 months (4, 53), implying that it might be a more suitable method for such patients in the current study. Furthermore, maintenance therapy seems to have no obvious survival benefit (54–56), but it is also worth trying (57, 58). In addition, among patients with the extensive stage, brain metastasis accounted for 53%. Oikawa et al. (59) analyzed the probability of distant metastasis sites of lung cancer and found that there was a specific pattern of lung cancer distant metastasis, that was when one site had metastasis, there was another subsequent site with a relatively high probability of metastasis (59). From the data of this study, there may be a similar relationship between parotid metastasis and brain metastasis of lung cancer. In other words, patients with lung cancer parotid metastasis seem to show a greater tendency of brain metastasis. Previous studies concluded that WBRT (60, 61), stereotactic radiosurgery (SRS) (62, 63), and prophylactic cranial irradiation (PCI) (62, 64, 65) can improve the prognosis of these patients and bring survival benefits. Therefore, for patients for whom brain metastasis already appears, WBRT or SRS should be performed as soon as possible. More importantly, for patients who have not yet developed brain metastasis, PCI should be applied in time (62). Furthermore, several studies (66, 67) have shown that parotidectomy seems to have no improvement in the prognosis of metastatic parotid tumors. However, for patients with parotid pain, parotidectomy is helpful to ameliorate their quality of life (30, 68).

### Outcome

Only a few studies reported the survival time in the current study, and the prognosis of SCC and AC was better than that of SCLC. Even after multiple treatments, the survival of SCLC patients with parotid metastasis is still short. Notably, as shown in present case 3, initially diagnosed at an early stage, the SCLC patient experienced a long disease-free survival after operation, but the condition deteriorated rapidly once parotid gland metastasis occurred. The case revealed the consistent viewpoint put forward by many previous studies that lung cancer parotid metastasis may be a marker of poor prognosis (13, 18, 24).

## Limitations

Our study has several limitations. First, due to the retrospective nature of the present study, the credibility needs to be verified. Second, for the rarity of parotid metastasis of lung cancer, the number of cases included in the current study is small; Owing to the superficial location and benign impression of parotid tumors, misdiagnosis of metastatic parotid tumors occurs quite a few, which may be another reason for the small samples; Moreover, we were unable to obtain the detailed clinical information of patients from some retrospective studies on secondary parotid metastasis, so these data were excluded from this study. Thus the current conclusion needs to be confirmed by a larger multicenter study. Third, the medical history and relevant data provided in many included cases are limited, and we cannot identify the clinical features, imaging features and survival time more specifically. Last, the metastasis of parotid gland in case 2 was not confirmed by pathological biopsy and was only a clinical diagnosis. Therefore, the value of the current study needs to be further inspected by prospective clinical research.

## Conclusions

The lung seems to be the most common primary site of parotid metastases except for head and neck tumors. Therefore, when secondary parotid malignancy is suspected and primary lesions are not found at the head and neck, the possibility of lung origin should be considered first. Clinical features, such as sex, age, smoking history, pathological types, and metastasis patterns, could provide valuable evidence for diagnosis. The two circumstances, SCLC coexisting with Warthin’s tumor and parotid small cell carcinoma with lung metastasis, should be differentiated from parotid metastasis of lung cancer with caution. For cases presented as SCLC, more aggressive strategies, such as chemotherapy with immunotherapy and maintenance therapy, may be more suitable. Due to the greater tendency of brain metastasis in such disease, WBRT, SRS or PCI should be applied to corresponding patients in time. Additionally, lung cancer parotid metastasis may be a marker of poor prognosis.

## Data availability statement

The datasets for this article are not publicly available due to concerns regarding participant/patient anonymity. Requests to access the datasets should be directed to the corresponding author.

## Author contributions

(I) Guarantor of integrity of the entire study: QZ; (II) Study concepts and design: QZ; (III) Literature research: RW, TW; (IV) Clinical studies: RW, TW; (V) Experimental studies/data analysis: RW, TW; (VI) Statistical analysis: RW, TW; (VII) Manuscript preparation: All authors; (VIII) Manuscript editing: All authors. All authors contributed to the article and approved the submitted version.

## Funding

This work was supported by the Regional Innovation Cooperation Project of Sichuan Science and Technology Program, China (2021YFQ0029).

## Conflict of interest

The authors declare that the research was conducted in the absence of any commercial or financial relationships that could be construed as a potential conflict of interest.

## Publisher’s note

All claims expressed in this article are solely those of the authors and do not necessarily represent those of their affiliated organizations, or those of the publisher, the editors and the reviewers. Any product that may be evaluated in this article, or claim that may be made by its manufacturer, is not guaranteed or endorsed by the publisher.
